# Leptospirosis presenting as acute acalculous cholecystitis

**DOI:** 10.1002/ccr3.1173

**Published:** 2017-09-15

**Authors:** Peter Davies, Yuki Aoyagi

**Affiliations:** ^1^ Medical Department Whangarei Base Hospital Whangarei New Zealand

**Keywords:** Acalculous cholecystitis, leptospirosis, pancytopenia, zoonoses

## Abstract

Leptospirosis is the commonest zoonotic infection worldwide but is vastly underreported and extremely heterogeneous in its presentation. Acalculous cholecystitis is an under recognized presentation of acute leptospirosis. In the appropriate clinical context, with a clear exposure history, recognition of this association presents a unifying diagnosis and limits unnecessary surgical interventions.

## Introduction

Leptospirosis is the most common zoonotic infection worldwide, with considerable morbidity and mortality. Reliable epidemiology remains elusive given the difficulty in establishing both a clinical and a serological diagnosis. Estimates from the World Health Organisation (WHO) suggest a worldwide incidence of 1.03 million cases per year with up to 58,000 deaths 1. Leptospirosis occurs worldwide, although it is more common in tropical areas, and is transmitted by a variety of mammalian vectors through direct or indirect exposure to urine or aborted tissues of infected animals. Leptospira encompass twenty‐two distinct species of spirochetes of which nine are pathogenic. Each species is adapted for a specific mammalian host, has different characteristics and complex virulence factors 2.

Acute leptospirosis is extremely heterogeneous in its presentation and can vary from a mild nonspecific self‐limiting febrile illness to organ dysfunction encompassing hepatitis, myocarditis, acute renal failure or pulmonary hemorrhage. A fulminant form, the so‐called Weil's disease presents with progressive jaundice and renal failure. The elusive unifying diagnosis of these disparate clinical findings is compounded by the reliance on expensive and time consuming confirmatory laboratory tests which are unfeasible in resource‐limited tropical locations. Indeed the majority of cases will be misdiagnosed as malaria, dengue, or other acute febrile illnesses. This constellation has contributed toward its status as a neglected tropical disease 3.

It is therefore important to be mindful of leptospirosis in those presenting with a nonspecific febrile illness, with or without any organ dysfunction, and to take an appropriate exposure history. In this study, we will discuss a case of leptospirosis presenting as acute acalculous cholecystitis (AAC) that with improved recognition this could present as a useful diagnostic sign of leptospirosis.

## Case History and Examination

A forty‐six‐year‐old woman presented to the emergency department with a six‐day history of a flu‐like illness encompassing malaise, myalgia, arthralgia, anorexia, and headaches without overt meningism. She reported episodes of fevers and rigors at home. She had developed persistent right upper quadrant pain the 2 days preceding her presentation, without change to bowel/bladder habits.

From a social perspective, she lives next to dairy farm and had recently visited the milking sheds. Her house is situated by a river from which she had exposure to during winter flooding. Furthermore, she had been recently exposed to rodents having removed a mouse from a trap a week beforehand. She is a nonsmoker and drinks up to 12 units of alcohol weekly. There was no relevant family or recent travel history.

On examination, she was afebrile with regular tachycardia at 112 bpm, blood pressure 109/60 mmHg, and respiratory rate 18 per minute. She appeared uncomfortable with nausea and abdominal pain. There was no evidence of jaundice, lymphadenopathy, or rash. Her heart sounds were dual, nil added, and her chest was clear. On abdominal examination, she demonstrated marked right upper quadrant tenderness with a positive Murphy's sign. She had no organomegaly or palpable masses.

## Differential Diagnosis

The history of a typical viral prodrome suggests perhaps influenza, cytomegalovirus (CMV), Ebstein–Barr virus (EBV), or an acute viral hepatitis. An acute hepatitis may have accounted for the abdominal discomfort. The exposure history to both a farm environment and domestic puts her at risk of leptospirosis. Finally, from a surgical perspective this may represent a simple acute calculous cholecystitis.

## Investigations and Treatment

Basic hematology revealed a mild pancytopenia with a hemoglobin of 116 mg/dL, leukocytes 2.0 per mm^3^ (neutrophils 68%, lymphocytes 14%), and platelets of 84 per mm^3^. Biochemistry demonstrated hypokalemia at 3.0 mmol/L and significant mixed liver dysfunction; alkaline phosphatase 296 U/L and alanine transferase 307 U/L. C‐reactive protein was elevated at 110 mg/L, and Albumin was 24 g/L on presentation.

**Figure 1:**
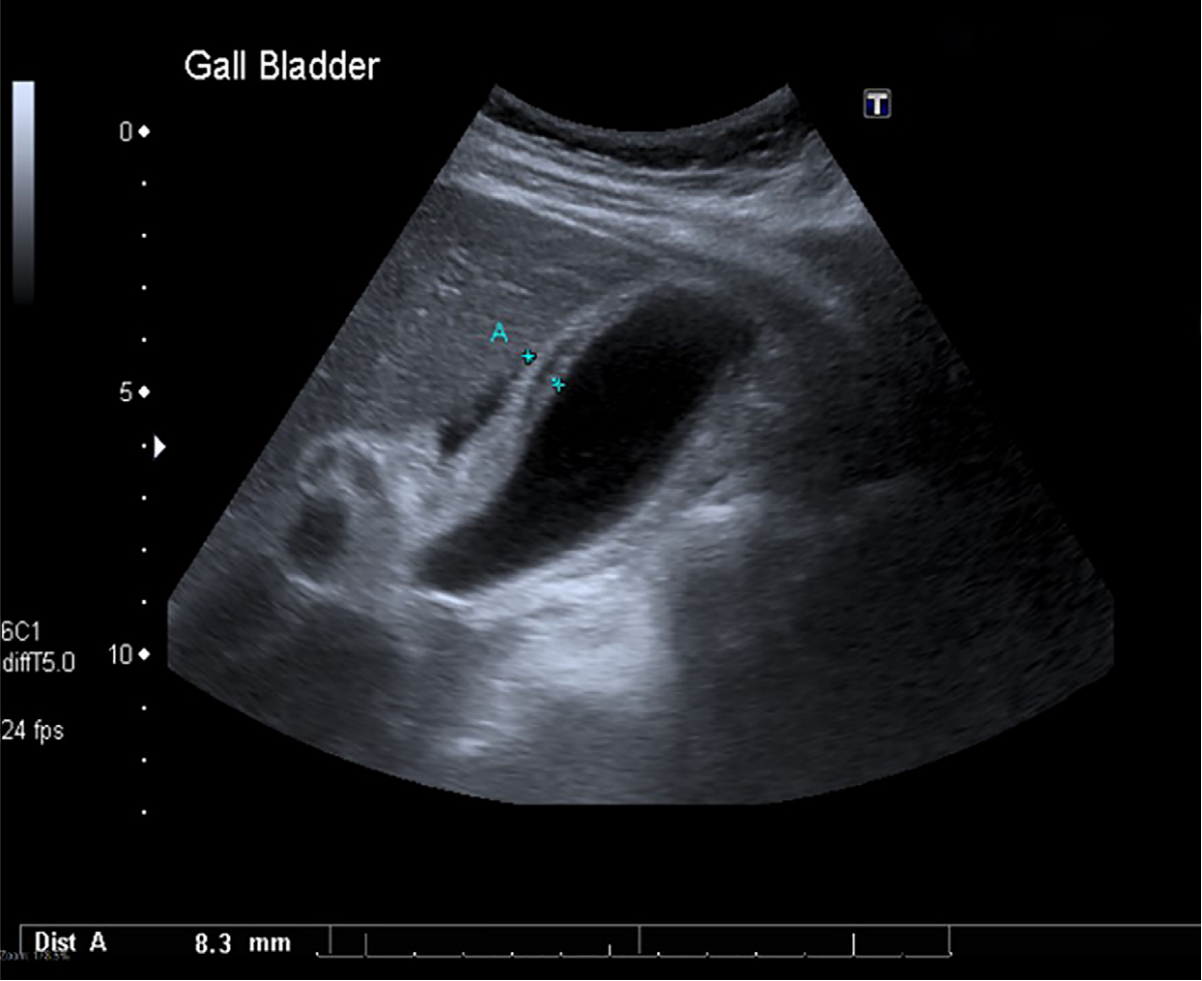
Cholecystitis with thickened and oedematous gallbladder wall, without evidence of cholelithiasis.

An abdominal ultrasound scan (USS) demonstrated a grossly edematous and thickened gallbladder wall measuring 8.3 mm, without sludge or calculi, which was consistent with an acute acalculous cholecystitis (Fig. 1). A chest X‐ray was normal.

Given her presentation, exposure history, and blood panel, she was treated empirically for leptospirosis with oral doxycycline and best supportive medical therapy. On surgical consultation, her antibiotics were switched to cefuroxime and metronidazole, and a magnetic resonance cholangiopancreatography (MRCP) was performed demonstrating no evidence of calculous disease.

## Outcome and Follow up

The patient gradually improved from both a clinical and a biochemical perspective over a three‐day period. She was discharged 4 days after presenting to the emergency department. She had negative serology for EBV, CMV, and HIV.

The diagnosis of leptospirosis was confirmed with dynamic titers to *Leptospira borgpetersenii* serovars *ballum/hardjo* with antibody levels rising from <25 to 1:800 over a two‐week period. This was thought to represent a co‐infection or cross reactivity between the serovars (a repeat titer over a longer interval may have been to differentiate the causative serovar). A full clinical and biochemical improvement was seen into the outpatient period, where she attended her general practitioner for follow‐up.

## Discussion

The highest prevalence of Leptospirosis is in the tropics yet its distribution includes temperate countries worldwide. It has a wide variety of vectors including rodents, cattle, swine, and in New Zealand hedgehogs and possums. The majority of cases relate to farm work with exposure to both vermin and domestic animals. Animal vaccination can be an effective way in achieving ruminant herd immunity to endemic serovars but remains imperfect with persistent low levels of urinary shedding 4. Clinical suspicion should be aroused for almost any presentation with a flu‐like prodrome, with or without organ dysfunction, in the context of environmental exposure. The constellation of “flu‐like” symptoms of fever, rigors, myalgia, and headache is seen in the vast majority of cases. More specific are the findings of conjunctival suffusion, hemorrhage (especially pulmonary), sterile pyuria, bilateral enlarged kidneys on USS, and aseptic meningitis 5. These variable and disparate signs and symptoms are due to an underlying vascular and endothelial injury (a vasculitis) that is induced by the leptospira. To this end almost all organs systems can be affected 6. AAC is considered as an organ specific example of this vasculitic process and is likely significantly underreported in leptospirosis.

The diagnosis of AAC is by ultrasound. Primarily this is based on a thickened gallbladder wall in the absence of calculi or sludge. A 3.5 mm cutoff for normal wall thickness is appropriate, producing a sensitivity of 80% and a specificity of 99% (a cutoff of 3 mm produces 100% sensitivity but is less specific). Other features include the presence of pericholecystic fluid (Fig. 1), emphysematous gallbladder wall, a striated gallbladder, and a positive sonographic Murphy's sign 7. AAC is more commonly seen in critical care patients of any underlying cause and conveys a high mortality. However, there are a variety of other infectious agents that make up a broad differential 8. These include viruses (EBV/CMV/advanced HIV), bacteria (Salmonella *enterica/typhi*), and parasites (*cryptosporidium*), and clinical suspicion is dependent on presentation and exposure history.

There have been a total of 14 reported cases of leptospirosis presenting with acute acalculous cholecystitis since 1993, with a typical demographic of working age males in developing countries, see Table 1 9, 10, 11, 12, 13, 14, 15, 16, 17, 18, 19. Co‐existing pancreatitis was described in 25% of reported cases with one death and one admission to intensive care 9, 10, 11, 12. The incidence of AAC in leptospirosis can be inferred from a mass exposure of 876 people following a triathlon in the United States, with 75 confirmed cases of leptospirosis, from which 2.6% (two confirmed cases) presented symptoms consistent with an acute cholecystitis 19. Beyond normal supportive treatment there was a high acute operative rate; 36% undergoing an emergency cholecystectomy. Yet interestingly a clinical presentation of severe cholecystitis contrasted with relatively mild findings on histology which demonstrated mild nonspecific pauci‐immune inflammation (with diagnostic intramural spirochetes) 19. Best supportive care with antibiotic treatment may therefore be preferable, with less operative risk in these acutely unwell patients. Furthermore, better recognition of this clinical presentation given the likely diagnosis (with exposure and prodrome) would ultimately limit unnecessary investigations; in this case a MRCP and a liver screen.

**Table 1:** Demonstrates total reported cases of leptospirosis associated with AAC in the literature with associated case characteristics 9, 10, 11, 12, 13, 14, 15, 16, 17, 18, 19

	Country	Sex	Exposure	Age	Species	Treatment	Complications
1	Brazil	M	Forest region	19	–	Antibiotics	Pneumonia, pericarditis
2	Turkey	F	–	40	*Interrogans* Icterohaemorrhagiae	Antibiotics	–
3	Thailand	M	Farmer	75	–	Cholecystectomy	–
4	Thailand	M	Farmer	36	–	Antibiotics	–
5	Thailand	M	Gardener	58	*Interrogans*	Antibiotics	–
6	Malaysia	M		83	–	Antibiotics	Pancreatitis
7	Japan	M	Farmer	66	*Interrogans* Autumnis	Antibiotics	Pancreatitis
8	USA	M	Triathlon	60	–	Cholecystectomy	–
9	USA	F	Triathlon	29	–	Cholecystectomy	–
10	Greece	M		21	*Interrogans*		Pancreatitis
11	India	M	Manuel Labourer	30	–	Antibiotics	Weil's disease
12	Singapore	M	Rat exposure	41	–	Antibiotics	Pancreatitis/Renal failure/ICU
13	Spain	M	Farmer	55	*Interrogans*/*Borgpetersenii* Hardjo	Cholecystectomy	Death
14	Martinique	M	–	36	*Interrogans* Icterohaemorrhagiae	Cholecystectomy	–

Where no information was available the field has been left empty.

The biochemical and hematological panel in this case demonstrates both typical and atypical findings in leptospirosis. Hypokalemia is the most common biochemical finding with or without the presence of acute kidney injury (AKI). This may be related to inhibition of the K+/Na+ pump on the ascending loop of Henle. Leptospirosis‐related AKI is novel in this regard and is usually nonoliguric 20. Thrombocytopenia is observed in approximately 75% of patients at day five of illness. In tropical regions, this can provide further diagnostic uncertainty in the undifferentiated febrile patient, in that it can mimic arboviruses such as dengue fever 21. A marked lymphopenia represents a common early finding yet a decreased total white cell as demonstrated as in this case is unusual. Conversely, a normal or slightly elevated leukocyte count is observed in the vast majority of patients and indeed an overt leucocytosis on presentation is associated with increased mortality 22. Hemoglobin levels largely remain normal but may show mild reduction through the illness depending on the presence of haemorrhage. A mild pancytopenia as seen in this case is comparatively uncommon but has been observed in other case reports 23. Other biochemical factors may be elevated depending on the system involved and the severity observed, including liver function tests, lipase/amylase, or troponin.

Confirmation of the clinical diagnosis remains difficult. Typically, immune seroconversion on a microscopic‐agglutination testing (MAT) forms the basis of diagnosis. The WHO—Leptospirosis epidemiology burden group set laboratory‐confirmed criteria for diagnosis. Indeed, the patient described here meets these criteria twofold with a greater than fourfold increase in titer between acute/convalescent samples and a MAT ≥1:400 in a single/paired sample. Other “confirmed” criteria include isolation of pathogenic leptospira from a sterile site and detection in clinical samples by histological, histochemical, immunostaining, or polymerase chain reaction (PCR) 24. Although the current gold‐standard, MAT remains labor intensive, specialized and the results semi‐subjective depending on regional prevalence/serovar panel tested. Molecular testing such as PCR continues to evolve and may represent the future of diagnostics 25.

Leptospira serovars vary in their underlying characteristics, with species such as L. *Interrogans* surviving for long durations within an aquatic environment causing epidemics in floods, triathletes, or adventure races 19. Conversely, L. *borgpetersenii*, seen in this case, has been shown to be comparatively genetically fragile, unable to survive beyond the host instead relying on direct transmission from the vector 26. There has only been one other report of a *Borgpetersenii* species in AAC and that as a co‐infection with an *Interrogans* species. This is the first reported case to our knowledge of AAC associated with a L. *borgpetersenii* species as the sole pathogen. The significance of this is unclear, and it may simply be due to transmission characteristics of the pathogen, as discussed. Instead, L. *interrogans* serovars icterohaemorrhagiae and autumnalis are the causative pathogens in the vast majority of cases presenting with AAC. The pathogenicity of leptospira is complex between species and strains, it remains poorly understood. One study has suggested a significant association between one species, L. *Interrogans* icterohaemorrhagiae, and more severe disease; however, this has not been widely collaborated 27. Similarly, there could also be an association between serogroups and the development of a Jarisch‐Herxheimer reaction, with L.*interrogans* autumnalis implicated 28.

In terms of treatment, the evidence behind the use of antibiotics remains uncertain. A Cochrane review concluded there was insufficient quality data for firm recommendations, yet noted a nonsignificant trend to swifter resolution with antibiotic therapy 29. Typically, for mild disease doxycycline is used as a first‐line agent, with azithromycin an alternative. This approach allows coverage for a rickettsial illness that may not be readily differentiated 30. Amoxicillin is considered for young children/pregnant patients. The patient in this case was hemodynamically stable without evidence of significant organ failure and therefore received oral doxycycline and was admitted for observation. With severe disease, clinical instability or progressive organ dysfunction, intravenous penicillin or cephalosporins (cefotaxime/ceftriaxone) are comparable. It is important to bear in mind that gallbladder stasis secondary to ACC could precipitate a secondary infection and therefore covering with a cephalosporin allows broader coverage including both Leptospira and enteric organisms. In this case metronidazole was also utilized and this approach could be generalizable to any other cause of AAC 31.

This case highlights key clinical signs, symptoms, and findings, relating to variable organ dysfunction that can contribute toward a clinical diagnosis of acute leptospirosis. Improved recognition of these could reduce unnecessary investigations, surgical intervention, and complication rates. In addition to providing a further clinical tool when suspecting this diagnosis.

## Authorship

PD: worked as the registrar on the team treating the patient; identified the case, consented and was the primary author, researcher and contributor to the manuscript. YA: worked as the consultant heading the team involved in the patient's care; provided advice on scope and nature of the manuscript in addition to aid with editing the manuscript.

## Conflicts of Interest

The authors have no competing interests and nothing to declare.
